# Perioperative immune checkpoint inhibition in head and neck squamous cell carcinoma: a 10-year review

**DOI:** 10.37349/etat.2026.1002406

**Published:** 2026-10-19

**Authors:** Henry Freeman, Clayton Rawson, Nadir Al-Saidi, Brandon Lucke-Wold, Michael Karsy, Mehrdad Pahlevani

**Affiliations:** Fondazione Policlinico Universitario Agostino Gemelli IRCCS, Italy; ^1^College of Osteopathic Medicine, Michigan State University, East Lansing, MI 48824, USA; ^2^Noorda College of Osteopathic Medicine, Provo, UT 84606, USA; ^3^Covenant Healthcare College of Medicine, Central Michigan University, Mount Pleasant, MI 48859, USA; ^4^Department of Neurosurgery, College of Medicine, University of Florida, Gainesville, FL 32611, USA; ^5^Department of Neurosurgery, Michigan Medicine, University of Michigan, Ann Arbor, MI 48109, USA; ^6^Department of Neurosurgery, Jamaica Hospital Medical Center, Richmond Hill, NY 11418, USA

**Keywords:** biomarkers, chemoradiotherapy combination trials, immune checkpoint inhibition, perioperative therapy, resectable head and neck squamous cell carcinoma

## Abstract

Immune checkpoint blockade has transformed management of recurrent or metastatic head and neck squamous cell carcinoma (HNSCC), yet efforts to integrate immunotherapy into curative chemoradiation have failed to improve outcomes. We conducted a narrative review of English-language literature published between 2015 and 2025, searching PubMed/MEDLINE, Embase, Web of Science, and ClinicalTrials.gov for perioperative (neoadjuvant and/or adjuvant) immunotherapy studies in resectable HNSCC, and reviewed translational and cross-tumor neoadjuvant data relevant to perioperative trial design. Across early-phase and randomized perioperative trials, neoadjuvant programmed cell death 1 (PD-1)-based strategies were consistently feasible and induced pathologic tumor regression in a subset of patients. Protocol-defined, treatment-related surgical delays were uncommon; however, delays overall were more frequent with perioperative pembrolizumab in the phase III setting (38 vs. 10 participants in KEYNOTE-689; median time to surgery 3.0 vs. 1.9 weeks), so preserved surgical deliverability should not be equated with absence of scheduling impact. Phase II studies associated depth of pathologic response and immune-inflamed tumor features with improved disease-free outcomes. These findings informed phase III perioperative trials, culminating in KEYNOTE-689, which improved event-free survival without compromising surgical completion. In contrast, phase III trials evaluating concurrent PD-1/programmed death-ligand 1 (PD-L1) checkpoint blockade with definitive chemoradiotherapy showed no meaningful survival benefit. Collectively, current evidence supports perioperative pembrolizumab added to surgery and risk-adapted adjuvant therapy in selected patients with resectable locally advanced HNSCC, while other perioperative immune checkpoint inhibitor regimens remain investigational. This conclusion rests principally on a single positive phase III trial (KEYNOTE-689), and the June 2025 FDA approval is restricted to tumors with a PD-L1 combined positive score (CPS) of at least 1. The relative contributions of neoadjuvant priming, concurrent postoperative therapy, and prolonged adjuvant maintenance cannot be disentangled within the KEYNOTE-689 design, and no perioperative schedule can yet be designated optimal. Ongoing studies will refine biomarker-guided selection, sequencing, and duration.

## Introduction

Head and neck squamous cell carcinoma (HNSCC) remains a major global health burden despite advances in surgery, radiotherapy (RT), and systemic therapy [1]. In resectable locally advanced disease, standard care is primary surgery followed by risk-adapted adjuvant RT with or without cisplatin-based chemoradiotherapy (CRT), yet recurrence remains clinically significant, particularly in human papillomavirus (HPV)-negative tumors [2–5]. Immune checkpoint inhibitors (ICIs) are established in recurrent/metastatic HNSCC, but adding programmed cell death 1 (PD-1)/programmed death-ligand 1 (PD-L1) blockade to definitive CRT has not produced consistent survival benefit in unresected locally advanced disease, underscoring the importance of treatment timing and immune context [6–9].

These results have shifted attention to perioperative strategies, where checkpoint blockade is delivered before and after surgery while the primary tumor and regional immune architecture remain intact, potentially enabling immune priming before surgical and adjuvant therapy-associated immune disruption. Early window-of-opportunity and perioperative trials have demonstrated biologic activity and preserved surgical deliverability within protocolized pathways, culminating in phase III evidence supporting event-free survival (EFS) improvement with perioperative pembrolizumab integrated with surgery and risk-adapted postoperative therapy. The 2015–2025 evolution from metastatic PD-1 success to negative definitive RT/CRT-immunotherapy combinations and, ultimately, effective perioperative sequencing is summarized in Figure 1 [7, 8, 10].

**Figure 1:**
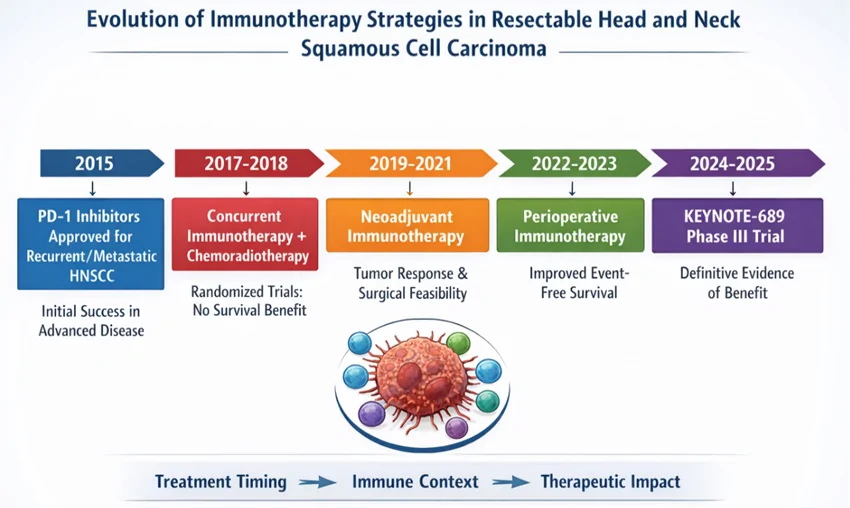
**Evolution of immunotherapy strategies in resectable head and neck squamous cell carcinoma (HNSCC).** Timeline illustrates the evolution of immune checkpoint inhibition strategies in HNSCC from 2015 to 2025.

This narrative review synthesizes the perioperative clinical trial landscape in resectable HNSCC, the biological rationale for neoadjuvant timing, the mechanistic and clinical context explaining failures of definitive CRT-immunotherapy strategies, and emerging biomarker frameworks that may enable response-adapted perioperative care. We performed a narrative review of the biologic rationale and clinical evidence for perioperative immunotherapy in resectable HNSCC. We searched PubMed/MEDLINE, Embase, and Web of Science for English-language articles published between January 1, 2015, and December 1, 2025. Search terms combined disease (HNSCC and relevant subsites); treatment context (neoadjuvant, adjuvant, perioperative, window-of-opportunity); immunotherapy agents/targets [PD-1, PD-L1, cytotoxic T-lymphocyte-associated antigen 4 (CTLA-4) and specific agents]; and outcomes/biomarkers (pathologic response, EFS, disease-free survival, overall survival (OS), PD-L1 combined positive score (CPS), HPV status, circulating biomarkers, and immune profiling).

To capture ongoing or recently reported studies, we also reviewed ClinicalTrials.gov and meeting abstracts from major oncology and head-and-neck conferences, and cross-referenced trial identifiers (e.g., national clinical trial numbers) when updated results were reported outside full-text publications. Because perioperative PD-1 strategies in HNSCC draw heavily on mechanistic insights from other solid tumors, we also reviewed landmark neoadjuvant immunotherapy trials and consensus statements in melanoma, non-small cell lung cancer (NSCLC), and esophageal/gastroesophageal junction (GEJ) cancer, as well as foundational work on RT-immunotherapy interactions and tumor microenvironment biology [11, 12]. Titles and abstracts were screened for relevance by two authors, followed by full-text review of eligible articles. We extracted trial phase and design, sample size, tumor site and HPV status, surgical resectability, treatment regimen and timing, primary and secondary endpoints, response metrics, survival outcomes, immune-related adverse events (irAEs), and surgical/toxicity outcomes, along with reported biomarker and translational findings. We identified 41 studies focusing on perioperative immunotherapy in resectable HNSCC and related salvage/neoadjuvant settings. The core perioperative and window-of-opportunity trials are summarized in Table 1, cross-trial themes are synthesized in Table 2, and trials evaluating definitive RT/CRT + ICI are summarized in Table 3.

**Table 1:** Key perioperative/window-of-opportunity immunotherapy trials in resectable HNSCC.

**Setting**	**Trial/Study**	**Phase & design**	**Population (*N*)**	**Regimen & timing**	**Primary endpoint(s)**	**Key efficacy results**	**Feasibility (surgery)**	**Safety (selected)**
**Frontline, resectable LA-HNSCC**	**KEYNOTE-689 [10] (perioperative pembrolizumab)**	Phase III; open-label randomized (pembro + SOC vs. SOC)	Resectable, LA-HNSCC (*N* = 714; randomized 363 vs. 351)	**Neoadj pembro** 200 mg q3w × 2 → surgery → **adjuvant RT ± cisplatin** (SOC) + **pembro** planned concomitantly with postop RT/CRT then continued (total 15 adjuvant cycles planned)	**EFS** sequentially in CPS ≥ 10, CPS ≥ 1, then total population	**EFS @36 mo:** CPS ≥ 10: 59.8% vs. 45.9% (HR 0.66; *P* = 0.004); CPS ≥ 1: 58.2% vs. 44.9% (HR 0.70; *P* = 0.003); total: 57.6% vs. 46.4% (HR 0.73; *P* = 0.008). **Path response after neoadj + surgery (pembro arm, total population):** MPR 9.4%, pCR 3.0%; **0%** in control by blinded review.	**Surgery completion:** 88.4% (pembro) vs. 87.7% (control). **Surgery delays:** 38 vs. 10; median time to surgery 3.0 vs. 1.9 weeks.	**Grade ≥ 3 TRAEs:** 44.6% vs. 42.9%; deaths due to AE 1.1% vs. 0.3%. Potentially immune-mediated AEs grade ≥ 3: 10.0% (pembro).
**Frontline, resectable HPV-unrelated/p16**	**Uppaluri et al., 2020 [13] (neoadj ± adjuv pembrolizumab)**	Phase II; multicenter	Resectable, LA **HPV-unrelated** HNSCC (*N* = 36)	**Neoadj pembro** 200 mg once → surgery 2–3 weeks later → planned postop (chemo) RT; **high-risk pathology** planned **adjuvant pembro** q3w × 6	Coprimary: **pTR-2 (≥ 50% regression)** (all pts) and **1-year relapse rate** in high-risk pathology	**pTR-2:** 22%; **pTR-1:** 22%; **any pTR ≥ 10%:** 44%; **pCR:** 0%. **1-year relapse rate** in high-risk: 16.7% (95% CI reported in paper).	Abstract reports **no unexpected surgical delays/complications**.	Abstract reports **no serious (grade 3–4) AEs** and no unexpected surgical delays/complications.
**Frontline, resectable p16 LA-HNSCC**	**Wise-Draper et al., 2022 [14] (peri-pembrolizumab)**	Phase II; open-label, nonrandomized; safety lead-in	Resectable HNSCC including **p16 oropharynx**; clinical stage high-risk features (*N* = 92)	**Neoadj pembro** 200 mg 7–21 days pre-op → surgery → adjuvant RT (60–66 Gy) + **pembro q3w × 6**; **high-risk** also got weekly cisplatin 40 mg/m^2^	**1-year DFS**	**1-year DFS:** 97% (intermediate-risk) vs. 66% (high-risk) at median follow-up 28 months. **Pathologic response** associated with improved 1-year DFS (93% vs. 72%; HR 0.29; 95% CI 0.11–0.77).	Feasibility assessed via **DLT defined as pembro-attributable delay** to SOC (> 7 days surgery, > 3 days RT, > 7 days cisplatin).	“No new safety signals” in abstract; DLT framework specified.
**Frontline, resectable HPV+/HPV−**	**CheckMate 358 [15] (neoadj nivolumab)**	Phase I/II; nonrandomized	Resectable HNSCC (*N* = 52; 26 HPV+, 26 HPV−)	**Nivolumab 240 mg** days 1 & 15 → surgery planned day 29 (± 7)	Primary: safety/tolerability; protocol-defined TRAE-related surgical delay **> 4 weeks**	No pCR by site/central review. **Centrally evaluable:** HPV+: MPR 5.9% + pPR 17.6% (= 23.5%); HPV−: pPR 5.9%.	**No protocol-defined TRAE-related surgical delay (> 4 weeks).**	**Grade 3–4 TRAEs:** 19.2% (HPV+) and 11.5% (HPV−).
**Frontline, oral cavity SCC**	**Schoenfeld et al., 2020 [16] (nivo vs. nivo + ipi)**	Phase II; open-label randomized (single center)	Untreated oral cavity SCC (*N* = 29; randomized 14 vs. 15)	**Nivolumab** 3 mg/kg weeks 1 & 3 ± **ipilimumab** 1 mg/kg week 1 → surgery 3–7 days after cycle 2	Coprimary: safety (incl surgical delays) and **volumetric response**	Evidence of response in both arms: volumetric response 50% vs. 53%; RECIST response 13% vs. 38%; pathologic response 54% vs. 73%. **Major/complete path response > 90%:** 1 (nivo) vs. 3 (combo).	**Median cycle 1 → surgery:** 19 days (range 7–21); **no surgical delays.**	Grade 3–4 events: 2 (nivo) vs. 5 (combo). One postoperative death judged unrelated to study treatment (reported in paper).
**Frontline (mostly) + some recurrent; major surgery planned**	**IMCISION [17] (nivolumab ± ipilimumab)**	Phase Ib/IIa; nonrandomized	Primary or recurrent resectable HNSCC (*N* = 32)	**2 doses**: nivolumab 240 mg weeks 1 & 3 ± **ipilimumab** 1 mg/kg week 1 → surgery week 5–6	Phase Ib: **feasibility** (resect ≤ week 6); Phase IIa: **pathologic response**	**MPR:** 17% (nivo mono) vs. 35% (combo) (reported overall/phase IIa as described in paper).	**Surgery not delayed or suspended** for any patient in phase Ib.	**Grade 3–4 irAEs:** 33% (nivo mono) vs. 38% (combo). **MPR definition** required ≤ 10% residual viable tumor **and** 90–100% decrease in viable tumor cells from baseline biopsy to on-treatment specimen (study’s conservative classification).
**Frontline, oral cavity SCC**	**Ju et al., 2022 [18] (camrelizumab + apatinib)**	Phase I; single-arm	LA resectable OSCC (*N* = 20)	**Camrelizumab** 200 mg q2w + **apatinib** 250 mg daily × 3 cycles → surgery	Primary: safety and **MPR (≤ 10% residual viable tumor)**	**MPR:** 40% (8/20). Post-hoc 18-month locoregional recurrence 10.5% and survival 95% (reported; follow-up limited).	One reported surgery delay (7 days) due to unexplained troponin elevation (resolved).	No neoadjuvant therapy-related grade ≥ 3 AEs reported in paper’s primary safety summary.
**Frontline, LA-HNSCC (China; mixed subsites)**	**Zhang et al., 2022 [19] (chemo + camrelizumab)**	Phase II; single-center single-arm	Resectable stage III–IVB HNSCC (*N* = 30; 27 underwent surgery)	**Chemo + camrelizumab** every 21 days × 3 → surgery (1–4 weeks after neoadj) → adjuvant RT	Coprimary: **pCR rate** and safety	**ORR:** 96.7% (29/30). **pCR:** 37.0% (95% CI 19.4–57.6). **MPR:** 74.1% (95% CI 53.7–88.9). **12-mo DFS:** 95.8% (95% CI 73.9–99.4).	**27/30 underwent surgery “without delay”;** R0 resection 92.6% (25/27).	Grade 3 neoadjuvant TRAEs: rash/pruritis/thrombocytopenia (each 3.3%); no grade 4/5. Surgical complication: delayed wound healing 18.5%.
**Salvage/Recurrent, resectable**	**Hanna et al., 2022 [20] (nivo + lirilumab peri-salvage)**	Phase II; single-arm	Recurrent, resectable HNSCC undergoing salvage surgery (*N* = 28; 96% prior radiation)	**Nivolumab 240 + lirilumab 240** once 7–21 days pre-op → salvage surgery → **6 cycles adjuvant** nivo + liri	Primary: **1-year DFS**	**Pathologic response:** 43% overall (MPR 14% [≤ 10% viability], PPR 29% [≤ 50% viability]); **1-year DFS:** 55.2%; **1-year OS:** 85.7% (reported).	**No delays to surgery** reported in abstract.	**Grade 3 + AEs:** 11% (reported).
**Salvage/recurrent, resectable**	**Leddon et al., 2022 [21] (adjuvant nivolumab post-salvage)**	Phase II; open-label; multi-institutional	Recurrent, resectable HNSCC post-salvage resection (*N* = 39)	Enroll ≤ 6 weeks post-op → **adjuvant nivolumab** planned for 6 cycles of 480 mg every 28 days (or equivalent dosing per protocol)	Primary: **2-year DFS** vs. historical	**2-year DFS:** 71.4% (95% CI 57.8–88.1). **2-year OS (from salvage surgery):** 77.7% (95% CI 64.1–94.2).	Not a neoadjuvant study (feasibility pertains to completing planned adjuvant therapy).	**Grade 3–4 TRAEs:** 7.7% (3/39); **8% discontinued** due to side effects; no treatment-related deaths reported during study follow-up.

Summary of key perioperative and window-of-opportunity immunotherapy trials in resectable HNSCC across frontline and salvage/recurrent settings. For each study, the table reports trial phase/design, population characteristics (including subsite and HPV/p16 enrichment when specified), perioperative regimen and timing (neoadjuvant window, surgery timing, and adjuvant therapy backbone), primary endpoint(s), and selected efficacy, feasibility, and safety outcomes. “Feasibility” is reported using each study’s stated definition (e.g., protocol-defined TRAE-related surgical delay thresholds vs. any delay), and “safety” summarizes selected grade ≥ 3 treatment-related and/or irAEs as reported. Pathologic response metrics (e.g., pTR categories, MPR, pCR, pPR) and their definitions vary across trials and are presented as defined in the original reports; therefore, cross-trial comparisons should be interpreted cautiously given heterogeneity in populations, endpoints, and reporting windows. AE: adverse event; CI: confidence interval; CPS: combined positive score; CRT: chemoradiotherapy; DFS: disease-free survival; DLT: dose-limiting toxicity; EFS: event-free survival; HNSCC: head and neck squamous cell carcinoma; HPV: human papillomavirus; HR: hazard ratio; irAE: immune-related adverse event; LA: locally advanced; MPR: major pathologic response; ORR: objective response rate; OS: overall survival; OSCC: oral squamous cell carcinoma; p16: p16 immunohistochemistry surrogate for human papillomavirus-associated disease; pCR: pathologic complete response; pPR: pathologic partial response; pTR: pathologic tumor response; q2w: every 2 weeks; q3w: every 3 weeks; RT: radiotherapy; SCC: squamous cell carcinoma; SOC: standard of care; TRAE: treatment-related adverse event.

**Table 2:** Cross-study synthesis of perioperative/window-of-opportunity immunotherapy in resectable HNSCC.

**Domain**	**Cross-study synthesis (manuscript-aligned wording)**	**Evidence basis (representative, not exhaustive)**
**Pathologic response (PD-1/PD-L1 monotherapy)**	Across neoadjuvant PD-1/PD-L1 **monotherapy** trials, **pCR is uncommon** and **MPR is modest** at the primary tumor site; pathologic response is best framed as an **indicator of immune engagement**, not a validated surrogate endpoint across all designs.	Masarwy et al. [22] pooled estimates across neoadjuvant PD-1/PD-L1 studies: **MPR 9.7%** and **pCR 2.9%** (primary tumor).
**Pathologic response (intensified neoadjuvant strategies)**	Intensified regimens (dual checkpoint blockade, chemoimmunotherapy, targeted combinations) can yield **higher short-term radiographic and pathologic response metrics** than monotherapy in selected cohorts, but cross-trial comparisons are limited by **heterogeneity in regimen, endpoints, and response definitions**, and combination approaches can narrow the perioperative safety margin.	Randomized dual ICI window trials report higher major/near-complete pathologic responses in combination arms with more grade ≥ 3 toxicity. Chemoimmunotherapy single-arm phase II studies show high ORR/MPR/pCR rates but require randomized confirmation. Zhu et al. [23] meta-analysis reports higher pooled response measures for chemoimmunotherapy vs. chemotherapy in included studies, with heterogeneity.
**Radiographic response**	Radiographic responses during short neoadjuvant windows are **variable** and can be **discordant** with pathologic regression; radiographic shrinkage should not be assumed to reflect immune-mediated pathologic response.	CheckMate 358 [15] reports radiographic reductions with limited linkage to pathologic response in available evaluable specimens; several window designs emphasize pathologic endpoints over radiographic response.
**Clinical benefit (survival endpoints)**	The strongest evidence for curative-intent benefit comes from **phase III perioperative strategy**. For neoadjuvant chemoimmunotherapy as a class, pooled survival conclusions remain **uncertain** due to heterogeneity and limited randomized long-term outcomes.	KEYNOTE-689 [10] demonstrates **EFS improvement** with perioperative pembrolizumab (overall HR reported in the trial). Zhu et al. [23] meta-analysis survival subgroup analyses do not establish a consistent OS/PFS advantage for chemoimmunotherapy vs. chemotherapy across included datasets.
**Feasibility (surgical timing)**	“Surgical delay” is **definition-dependent** (protocol-defined vs. any delay). Across many neoadjuvant ICI trials, protocol-defined immunotherapy-related delays are **uncommon**, but delays can occur—especially in extended perioperative regimens—even when overall surgical completion is similar between arms.	Masarwy et al. [22] reports very low pooled surgical delay signals in included neoadjuvant PD-1/PD-L1 studies. In contrast, KEYNOTE-689 [10] reports **more surgery delays** in the perioperative pembrolizumab arm despite similar overall surgical completion, highlighting the importance of specifying definitions and denominators.
**Safety (where toxicity “lands”)**	In pooled neoadjuvant PD-1/PD-L1 monotherapy experience, **preoperative grade 3–4 TRAEs are generally low**, but toxicity burden increases with **combination therapy** and with **extended perioperative regimens** (especially when layered onto adjuvant RT/CRT). Manuscript wording should specify **grade**, **attribution** (TRAE/irAE), and **treatment phase** (neoadjuvant vs. adjuvant).	Masarwy et al. [22] pooled preoperative **grade 3–4 TRAEs 8.4%**. Dual-checkpoint trials report higher grade ≥ 3 events than monotherapy. KEYNOTE-689 [10] reports substantial grade ≥ 3 TRAEs in both arms, with higher immune-mediated toxicity in the pembrolizumab arm and notable discontinuation rates—underscoring that “acceptable safety” depends on context and window.
**Biomarkers (signal vs. ready-for-prime-time)**	**PD-L1/CPS** is widely measured and used for stratification in large trials, but across smaller perioperative cohorts its association with pathologic response is **inconsistent**; it should be framed as a **contextual marker** rather than a universal predictor. Immune-inflamed/IFN-γ-related signatures and immune infiltration measures show **signals of association** in some studies, but remain **exploratory**.	KEYNOTE-689 [10] reports EFS by CPS strata (supports CPS as a stratification/selection framework at scale). Smaller perioperative cohorts show variable associations between PD-L1 and response; immune gene-expression/infiltration signals are frequently exploratory. Zhu et al. [23] emphasizes the need for biomarker-driven refinement and additional randomized validation.
**Evidence strength (how confident we should be)**	Evidence spans small window trials and phase II single-arm cohorts to **phase III perioperative** data. Meta-analyses provide pooled benchmarks but may include heterogenous designs and partially reported datasets, so synthesis should be presented as **supportive context**, not definitive proof.	Masarwy et al. [22] includes a limited number of heterogeneous neoadjuvant ICI studies (some abstract-level), useful for pooled feasibility/safety/pathologic response benchmarks. KEYNOTE-689 [10] provides the highest-level evidence for perioperative efficacy. Zhu et al. [23] meta-analysis underscores heterogeneity and the need for higher-quality randomized survival data.

Summary of cross-study patterns (patterns across studies, not individual trials) in perioperative and window-of-opportunity immunotherapy for resectable HNSCC. Rather than listing individual trials, it synthesizes recurring themes that inform clinical interpretation and trial design: (i) pathologic response behavior across treatment platforms, (ii) feasibility considerations with emphasis on how “surgical delay” is defined, (iii) safety signals stratified by treatment phase (neoadjuvant vs. extended perioperative/adjuvant), (iv) biomarker signals that are associative/exploratory versus clinically implementable, and (v) evidence strength across study designs (phase II vs. phase III; randomized vs. single-arm). Pooled benchmarks are drawn from systematic reviews/meta-analyses and interpreted alongside the phase III perioperative pembrolizumab trial (KEYNOTE-689) and representative perioperative cohorts included in Table 1. Because response definitions (e.g., MPR/pCR/pTR) and reporting windows vary across studies, synthesis statements are framed to avoid implying validated prediction unless supported by the underlying data. CPS: combined positive score; CRT: chemoradiotherapy; EFS: event-free survival; HNSCC: head and neck squamous cell carcinoma; ICI: immune checkpoint inhibitor; IFN-γ: interferon-gamma; irAE: immune-related adverse event; MPR: major pathologic response; ORR: objective response rate; OS: overall survival; pCR: pathologic complete response; PD-1: programmed cell death 1; PD-L1: programmed death-ligand 1; PFS: progression-free survival; pTR: pathologic tumor response; RT: radiotherapy; TRAEs: treatment-related adverse events.

**Table 3:** Definitive CRT/RT plus immune checkpoint inhibition trials in unresected locally advanced HNSCC.

**Trial (Identifier)**	**Phase & design**	**Population/Setting**	**Regimen & timing (experimental vs. control)**	**Primary endpoint**	**Primary endpoint result**	**Key feasibility/safety notes**
**JAVELIN Head & Neck 100** [7] (NCT02952586)	Phase III; randomized; double-blind; placebo-controlled	Newly diagnosed, **unresected** locally advanced HNSCC eligible for cisplatin-based CRT	**Avelumab** lead-in (single dose 7 days pre-CRT) + **concurrent** with cisplatin-CRT + **maintenance** q2w up to 12 months vs. placebo + CRT	**PFS** (investigator-assessed)	PFS **not improved**; stratified HR **1.21** (95% CI **0.93–1.57**), one-sided *P* = **0.92** (numerically favored placebo)	Common grade ≥ 3 TRAEs: neutropenia **16% vs. 15%**, mucosal inflammation **14% vs. 13%**, dysphagia **14% vs. 14%**, anemia **12% vs. 13%**. OS not reached; OS HR **1.31** (95% CI **0.93–1.85**), one-sided *P* = **0.94** (hierarchical testing limited after primary endpoint not met) (Lee et al., 2021 [7])
**KEYNOTE-412** [8] (NCT03040999)	Phase III; randomized; double-blind; placebo-controlled	Newly diagnosed, high-risk, **unresected** locally advanced HNSCC	**Pembrolizumab** q3w up to 17 doses (**1 pre-CRT**, **2 during CRT**, then **maintenance**) + cisplatin-CRT vs. placebo + CRT	**EFS**	EFS **did not meet prespecified significance**: HR **0.83** (95% CI **0.68–1.03**), *P* = **0.043** (threshold ***P* < 0.024**)	Grade ≥ 3 AEs: **92% vs. 88%**. OS at 36 months: **72% vs. 70%** (HR **0.90**, 95% CI **0.71–1.15**). AEs led to discontinuation of any study treatment: **41% vs. 33%** (Machiels et al., 2024 [8])
**GORTEC 2015-01** [9] **(PembroRad)** (NCT02707588)	Phase II; randomized	Locally advanced HNSCC **unfit for high-dose cisplatin**	**Pembrolizumab + RT** (concurrent) vs. **cetuximab + RT** (concurrent)	**LRC at 15 months**	LRC at 15 months: **60% vs. 59%**; OR **1.05** (95% CI **0.43–2.59**), *P* = **0.91**	No significant differences in PFS (HR **0.85**, 95% CI **0.55–1.32**, *P* = **0.47**) or OS (HR **0.83**, 95% CI **0.49–1.40**, *P* = **0.49**). Grade ≥ 3 AEs: **74% vs. 92%** (*P* = **0.006**). Rare but severe vascular toxicity reported in pembrolizumab-RT arm (including arterial thromboses and retinal artery occlusion) (Tao et al., 2023 [9])
**Weiss et al., 2020** [24] **(RT + pembrolizumab; cisplatin-contraindicated)** (NCT02609503)	Phase II; single-arm	Stage III–IV HNSCC **cisplatin-ineligible/contraindicated**	Definitive **RT + pembrolizumab** (concurrent × 3 doses, then adjuvant × 3 doses)	Primary: median PFS ≥ 16 months (target)	Primary endpoint met; median PFS **not reached** at median follow-up **21 months**; estimated **24-mo PFS 71%** (95% CI **49–84**) and **24-month OS 75%** (95% CI **51–88**)	High grade 3/4 lymphopenia: **58.6%** (Weiss et al., 2020 [24])

Summary of definitive (non-surgical) trials evaluating PD-1/PD-L1 immune checkpoint inhibitors combined with RT or cisplatin-based CRT in unresected locally advanced HNSCC. Across randomized phase III CRT platforms (JAVELIN Head & Neck 100; KEYNOTE-412) and cisplatin-ineligible phase II RT-based platforms (PembroRad; Weiss et al. [24]), adding immune checkpoint inhibition to definitive RT/CRT did not consistently improve primary survival or locoregional control endpoints in unselected populations. Feasibility and safety interpretation should account for backbone therapy (CRT vs. RT-only), eligibility (cisplatin-eligible vs. ineligible), and protocol-specific definitions of treatment interruption/discontinuation. AEs: adverse events; CI: confidence interval; CRT: chemoradiotherapy; EFS: event-free survival; HNSCC: head and neck squamous cell carcinoma; HR: hazard ratio; OS: overall survival; PD-1: programmed cell death 1; PD-L1: programmed death-ligand 1; PFS: progression-free survival; q2w: every 2 weeks; q3w: every 3 weeks; RT: radiotherapy; TRAEs: treatment-related adverse events.

This work is a narrative review, and the search and screening workflow described above should not be interpreted as a systematic review. Although titles and abstracts were screened by two authors with subsequent full-text review and structured data extraction, no protocol was registered or published, no PRISMA flow diagram was generated, no formal risk-of-bias appraisal of included studies was performed, and no meta-analysis was undertaken. The 41 studies identified reflect selective, purposive inclusion of the trials and translational datasets judged most informative for perioperative strategy in resectable HNSCC, rather than an exhaustive enumeration of all literature meeting predefined eligibility criteria. Quantitative pooling across these studies was not attempted and would not be appropriate given the heterogeneity in design, population, regimen, and endpoint definitions described throughout. This synthesis should therefore be read as an expert-curated overview that is subject to selection and publication bias, and its conclusions carry correspondingly lower evidentiary weight than those of a registered systematic review.

## Clinical trial landscape of perioperative immunotherapy in resectable HNSCC

Clinical evaluation of immune checkpoint inhibition in resectable HNSCC has progressed through a series of perioperative and window-of-opportunity trials designed to test whether immunotherapy delivered around surgical resection can improve curative-intent outcomes. Across this evolving landscape, neoadjuvant and perioperative PD-1-based strategies have consistently demonstrated feasibility, acceptable safety, and biologic activity, in contrast to prior attempts to integrate immune checkpoint inhibition concurrently with definitive CRT [10, 13–15].

Early signal-generating evidence emerged from single-arm phase II neoadjuvant PD-1 blockade studies. In a multicenter trial of patients with resectable, locally advanced HPV-unrelated HNSCC, neoadjuvant pembrolizumab administered prior to surgery was feasible, did not delay definitive resection, and induced measurable pathologic tumor regression in a substantial subset of patients [13]. Any pathologic response (≥ 10% tumor regression) was observed in approximately 44% of tumors, and pathologic response correlated with baseline PD-L1 expression, interferon-gamma (IFN-γ)-related gene-expression signatures, and intratumoral CD8^+^ T-cell infiltration, supporting a biologically meaningful immune effect of preoperative PD-1 blockade [13].

Subsequent phase II studies expanded this approach across broader HNSCC populations and perioperative regimens. Wise-Draper et al. [14] evaluated neoadjuvant pembrolizumab followed by surgery and adjuvant pembrolizumab administered with postoperative RT with or without cisplatin in patients with resectable, locally regionally advanced HNSCC. The authors confirmed that perioperative PD-1 blockade was safe, was associated with few protocol-defined treatment-attributable delays to surgery (that trial’s feasibility endpoint being a dose-limiting toxicity defined as a pembrolizumab-attributable delay of more than 7 days to surgery), and yielded quantifiable pathologic tumor regression that correlated with improved disease-free survival, supporting pathologic response as a clinically informative early endpoint, although not a validated surrogate for survival in HNSCC [14].

Neoadjuvant nivolumab was evaluated in both HPV-positive and HPV-negative resectable HNSCC cohorts in the CheckMate 358 trial [15]. Short-course preoperative nivolumab was feasible, did not compromise surgical timing, and resulted in pathologic tumor regression in a meaningful subset of patients, with higher rates observed in HPV-positive tumors [15]. In this context, the relevant implication is the potential advantage of administering PD-1 blockade before surgery, while the primary tumor and regional lymphatic immune context remain intact enough to support antitumor priming [15].

Across early-phase neoadjuvant PD-1/PD-L1 monotherapy trials, radiographic responses were uncommon, but pathologic responses emerged as a reproducible indicator of immune engagement and a potential early surrogate of clinical benefit [13–15]. At initial evaluation, the most relevant baseline immune-oncology assessment is pragmatic rather than expansive: tumor HPV/p16 status and PD-L1 expression, together with confirmation of resectability, clinical risk features, performance status, organ function, and exclusion of major contraindications to checkpoint blockade; by contrast, deeper profiling of CD8^+^ infiltrates, IFN-γ-related programs, or other immune signatures remains primarily investigational at present [13–15].

An explicit caution regarding pathologic endpoints belongs here rather than solely in the Limitations section. Unlike NSCLC and melanoma, in which neoadjuvant pathologic response has been extensively characterized and is increasingly accepted as an early efficacy readout, pathologic response in HNSCC is neither standardized nor validated as a surrogate for survival. Definitions differ across trials, with pathologic tumor response (pTR) categories, MPR, pathologic complete response (pCR), and pathologic partial response (pPR) applied using different viable-tumor thresholds; assessment may be performed by central or by local review; and the primary tumor and nodal disease may be scored jointly or separately. Validation as a survival surrogate has not been established across HPV status, anatomic subsite, or treatment platform, and the association between depth of pathologic regression and long-term outcome observed within individual cohorts has not been shown to hold across regimens. The associations between pathologic response and disease-free outcomes described in this section should therefore be read as internally consistent observations within individual trials, and cross-trial comparisons of response rates, including those tabulated in Tables 1 and 2, should be interpreted with corresponding caution.

Building on monotherapy experience, several early-phase studies explored intensified neoadjuvant strategies incorporating dual immune checkpoint blockades or combinations with cytotoxic or targeted agents. Dual PD-1 and CTLA-4 inhibition produced higher rates of major or near-complete pathologic response compared with PD-1 monotherapy, but these gains were accompanied by increased immune-related toxicity, narrowing the perioperative therapeutic window [16, 17]. Additional pilot studies evaluating neoadjuvant chemoimmunotherapy or immunotherapy combined with antiangiogenic agents reported encouraging radiographic and pathologic responses; however, heterogeneity in study design, treatment regimens, and endpoints limits cross-trial comparisons and currently confines these approaches to investigational settings [18, 19, 23].

Perioperative immunotherapy has also been evaluated in the salvage setting, where recurrence after definitive CRT confers high risk and limited postoperative treatment options. In a phase II trial of adjuvant nivolumab following salvage resection, treatment was feasible, associated with manageable toxicity, and resulted in disease-free survival outcomes exceeding historical expectations [21]. Complementary evidence from a phase II study of neoadjuvant and adjuvant nivolumab combined with lirilumab in patients undergoing salvage surgery demonstrated pathologic tumor regression in a subset of patients and encouraging survival outcomes without compromising surgical feasibility [20].

The most definitive evidence supporting perioperative immunotherapy in resectable HNSCC derives from the randomized phase III KEYNOTE-689 trial [10]. In this study, patients with resectable stage III-IVA HNSCC were randomized to standard-of-care surgery followed by risk-adapted postoperative RT with or without cisplatin, or to the same standard-of-care approach plus perioperative pembrolizumab administered as two neoadjuvant cycles followed by postoperative pembrolizumab given concurrently with RT or CRT and then continued in the adjuvant setting [10]. Perioperative pembrolizumab significantly improved EFS across prespecified PD-L1 CPS strata, including the intention-to-treat population, and surgical completion rates were similar between groups. It is important to distinguish the trial population from the regulatory indication: although EFS benefit was demonstrated in the total randomized population, the June 2025 FDA approval of perioperative pembrolizumab is restricted to resectable locally advanced HNSCC with CPS ≥ 1. Patients with CPS-negative tumors therefore fall outside the approved indication despite having been represented in the trial, and this distinction should be made explicit whenever broad adoption is discussed [10]. This point is central to the timing theme of this review and warrants emphasis rather than relegation to a caveat: because KEYNOTE-689 tested an integrated perioperative package against standard of care, the relative contributions of neoadjuvant priming, concurrent postoperative therapy, and prolonged adjuvant maintenance cannot be disentangled within this trial design. The trial therefore establishes that the perioperative package works; it does not establish which component, or which sequence, is responsible for benefit. Accordingly, this regimen should be viewed as the current best-supported perioperative approach rather than a definitively optimized one, and claims that neoadjuvant priming plus adjuvant maintenance represents the optimal configuration remain hypotheses generated by, not conclusions from, this dataset [10]. In addition, although no new safety signals were identified, surgery delays were more frequent in the pembrolizumab arm, underscoring that perioperative immune benefit must still be balanced against practical recovery and wound-healing considerations when integrating treatment around surgery. It should also be noted that EFS, not OS, was the primary endpoint of KEYNOTE-689, and that OS data were not mature at the time of the primary report and at the time of this submission; conclusions regarding a survival advantage therefore remain provisional pending longer follow-up [10].

## Biological and translational rationale for perioperative timing

Preclinical work supports the concept that when checkpoint blockade is delivered relative to surgery can meaningfully influence systemic immune control of micrometastatic disease. In murine models of spontaneously metastatic breast cancer, neoadjuvant immunotherapy administered before primary tumor resection demonstrated significantly greater therapeutic efficacy than the same therapies delivered exclusively after surgery. This effect was attributed to elevated and sustained peripheral tumor-specific immune responses while tumor antigen remained abundant, and early post-treatment blood sampling of tumor-specific CD8^+^ T cells was proposed as a potential biomarker of outcome in these models [25]. This perioperative “priming then maintenance” framework is schematized in Figure 2.

**Figure 2:**
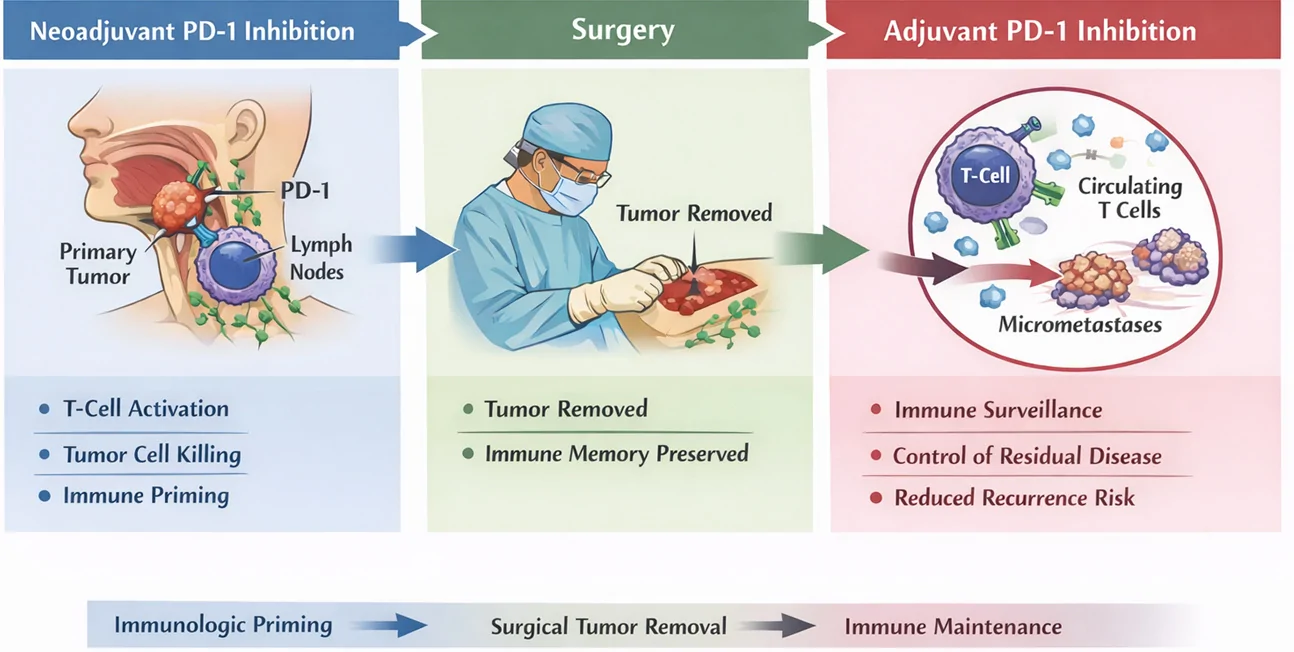
**Biologic rationale for perioperative immune checkpoint inhibition in resectable head and neck squamous cell carcinoma.** Conceptual model illustrating the biologic rationale for perioperative immune checkpoint inhibition in resectable head and neck squamous cell carcinoma. Neoadjuvant programmed cell death 1 (PD-1) blockade leverages the intact primary tumor and regional lymphatic network to prime and expand tumor-specific T-cell populations. Surgical resection removes the primary tumor while preserving systemic immune memory generated during neoadjuvant therapy. Postoperative immunotherapy sustains immune surveillance during the period of highest recurrence risk, enhancing control of occult micrometastatic disease. Together, this strategy integrates immunologic priming, surgical cytoreduction, and immune maintenance to optimize antitumor immunity.

Human translational studies in other solid tumors further support this timing-dependent framework. In resectable NSCLC, neoadjuvant PD-1 blockade was associated with major pathologic responses in a subset of patients and expansion of T-cell clones detectable in both tumor and peripheral blood, consistent with systemic mobilization of tumor-reactive immunity during the presurgical window [26].

Mechanistically, neoadjuvant PD-1/PD-L1 blockade is proposed to enhance systemic antitumor immunity through two nonmutually exclusive pathways: (1) reinvigoration and expansion of tumor-specific cytotoxic T cells already present within the tumor microenvironment, and/or (2) enhanced antigen presentation and productive T-cell priming in tumor-draining lymph nodes followed by trafficking of activated T cells to micrometastatic sites [27–29]. Both models emphasize the biologic advantage of delivering checkpoint blockade while the primary tumor and associated antigen-draining lymphatic structures remain intact.

In HNSCC, emerging single-cell and spatial profiling data suggest that early response to neoadjuvant anti-PD-1 therapy may be driven largely by activation of pre-existing, tissue-resident memory-like cytotoxic CD8^+^ tumor-infiltrating lymphocytes (e.g., CTX^+^/ZNF683^+^ programs), whereas nonresponding tumors show relative depletion of these populations and accumulation of more exhausted states [30]. These observations support a perioperative rationale in which neoadjuvant PD-1 blockade leverages the intact tumor-immune interface to amplify tumor-reactive immunity before surgical extirpation, while postoperative immunotherapy may help sustain and consolidate systemic immune control during a period of high recurrence risk [13, 14].

## Why concurrent CRT-immunotherapy strategies failed in locally advanced HNSCC

Despite strong preclinical rationale and clear efficacy of PD-1/PD-L1 blockade in recurrent or metastatic HNSCC, multiple phase III trials evaluating the addition of immune checkpoint inhibition to definitive cisplatin-based CRT in locally advanced disease have failed to demonstrate meaningful survival benefit. These negative results highlight the importance of treatment timing and immune context and provide critical contrast to the success of perioperative immunotherapy strategies.

Two large, randomized phase III trials, JAVELIN Head & Neck 100 and KEYNOTE-412, tested whether layering PD-1/PD-L1 blockade onto standard definitive CRT could improve outcomes in patients with unresected or locally advanced HNSCC. In JAVELIN Head & Neck 100, patients were randomized to receive cisplatin-based CRT with avelumab administered as lead-in, concurrent, and maintenance therapy versus CRT alone. The trial was terminated early for futility after interim analysis demonstrated no improvement in progression-free survival, with hazard ratios numerically favoring the control arm and no clear OS benefit observed [6, 7]. Similarly, KEYNOTE-412 evaluated pembrolizumab added to definitive CRT, delivered before, during, and after radiation, and failed to meet its prespecified EFS endpoint in the overall population, despite numerical trends favoring pembrolizumab in exploratory subgroup analyses [8]. The major definitive RT/CRT plus immune checkpoint inhibition trials are summarized in Table 3, and the published hazard ratios contrasting these trials with perioperative sequencing are shown as a forest plot in Figure 3.

**Figure 3:**
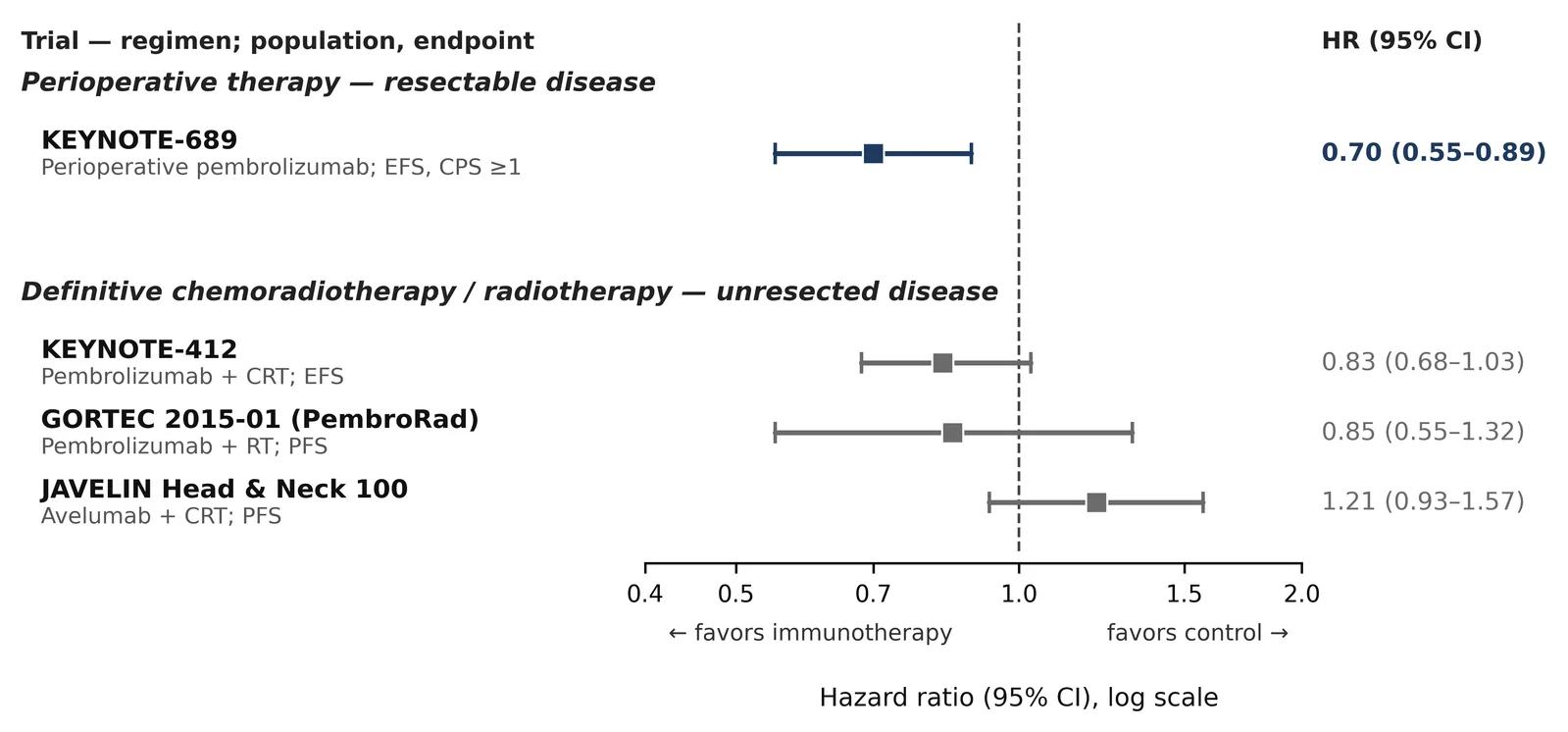
**Forest plot of published hazard ratios for immune checkpoint inhibition in locally advanced head and neck squamous cell carcinoma, contrasting the positive perioperative trial with the negative definitive CRT and RT trials.** Squares are point estimates and horizontal lines are 95% CIs, drawn directly from the trials summarized in Tables 1 and 3. KEYNOTE-689 evaluated perioperative pembrolizumab in resectable disease and significantly improved EFS [HR 0.70 (95% CI 0.55–0.89) in the PD-L1 CPS ≥ 1 population; the CPS ≥ 10 and total populations were also significant, with hazard ratios of 0.66 and 0.73, respectively]. In contrast, KEYNOTE-412 (EFS), GORTEC 2015-01/PembroRad (PFS), and JAVELIN Head & Neck 100 (PFS) added checkpoint blockade to definitive CRT or RT in unresected disease and did not demonstrate a significant benefit; their CIs cross a hazard ratio of 1.0, and JAVELIN Head & Neck 100 numerically favored the control arm. Because these trials differ in design, population, and endpoint definition, each row is annotated with its regimen, population, and endpoint, and no pooled summary estimate is shown; the figure is a qualitative contrast of published effect sizes and is not a meta-analysis. Hazard ratios below 1.0 favor the immunotherapy-containing regimen.CI: confidence interval; CPS: combined positive score; CRT: chemoradiotherapy; EFS: event-free survival; HR: hazard ratio; PD-L1: programmed death-ligand 1; PFS: progression-free survival; RT: radiotherapy.

Earlier phase II studies in cisplatin-ineligible populations also suggested that concurrent immunoradiotherapy may be feasible but raised concerns regarding immune suppression. In a single-arm phase II study of definitive RT with concurrent and adjuvant pembrolizumab, encouraging disease control was observed; however, a high incidence of grade 3–4 lymphopenia was reported, underscoring a potential mechanistic barrier to effective immune engagement in this setting [24]. The randomized phase II GORTEC 2015-01 (PembroRad) trial similarly demonstrated no improvement in locoregional control, progression-free survival, or OS with pembrolizumab-RT compared with cetuximab-RT, despite a more favorable toxicity profile in the pembrolizumab arm [9].

Several mechanisms have been proposed to explain the limited efficacy of concurrent CRT-immunotherapy strategies in HNSCC. The following account should be read as hypothesis-generating rather than established: these trials may have failed for multiple, non-exclusive reasons, including patient selection, PD-L1 enrichment strategy, CRT-induced lymphopenia, elective nodal irradiation, competing toxicity, insufficient treatment duration, suboptimal sequencing, and inherent biological differences between unresected and resectable disease. No single mechanistic explanation has been definitively validated in humans, and the mechanisms below are largely inferred from preclinical models and correlative analyses rather than from prospective mechanistic testing within the negative trials themselves. Treatment-induced lymphopenia has been proposed as one such barrier. In patients receiving definitive chemoradiation for HNSCC, severe lymphopenia develops in a substantial proportion of patients and has been independently associated with inferior progression-free survival, particularly in HPV-negative disease [31]. Across solid tumors, RT field size and dose distribution strongly influence the severity and duration of lymphocyte depletion, with larger treated volumes and elective nodal irradiation contributing to profound and prolonged immune suppression [32].

Beyond systemic lymphopenia, irradiation of tumor-draining lymph nodes appears particularly detrimental to checkpoint inhibitor efficacy. Preclinical studies demonstrate that elective nodal irradiation attenuates systemic antitumor immune responses and diminishes the combinatorial benefit of RT and PD-1 blockade by disrupting antigen presentation and T-cell priming within draining lymphatic basins [33, 34]. Additional work has shown that the sequence of PD-1 blockades relative to local irradiation critically determines the magnitude and breadth of systemic immune responses, further emphasizing the importance of timing [35].

The intrinsic immunobiology of HNSCC may further amplify these putative treatment-related barriers, although this too remains a working hypothesis. HNSCC tumors are characterized by highly immunosuppressive microenvironments enriched with myeloid-derived suppressor cells, tumor-associated macrophages, regulatory T cells, and dense stromal architecture that restricts effective T-cell infiltration and function [11, 36]. While RT can induce immunogenic cell death and increase antigen release, it can also exacerbate immune suppression depending on dose, fractionation, and target volumes, particularly when combined with treatment-related cytopenias [12, 37]. Systemically, such cytopenias may reduce the circulating pool of effector lymphocytes and supportive immune subsets available for antigen presentation, priming, and trafficking, thereby weakening antitumor immune surveillance beyond the irradiated field and favoring durable immune escape. In parallel, selective depletion of radiosensitive immune populations may leave relatively radioresistant suppressive populations, including regulatory T cells and myeloid-derived suppressor cells, proportionally enriched, further shifting the host response away from effective tumor control [11, 12, 24, 36, 37].

## HPV-positive and HPV-negative disease: stratification in perioperative strategy

HPV-positive and HPV-negative HNSCC are biologically and clinically distinct entities, differing in mutational burden, immune infiltration, prognosis, and pattern of failure. Much of the synthesis above necessarily treats them together, because most perioperative trials were neither powered nor prospectively stratified to resolve them; that pooling is a limitation of the evidence base rather than a reflection of shared biology, and several stratification questions deserve explicit statement.

Whether pathologic response rates differ meaningfully by HPV status remains incompletely resolved. CheckMate 358 reported numerically higher pathologic regression in HPV-positive than HPV-negative tumors among centrally evaluable specimens, consistent with the more immune-infiltrated phenotype typical of HPV-associated disease. However, the trials that most directly informed perioperative practice were weighted toward HPV-unrelated disease: the Uppaluri phase II trial enrolled HPV-unrelated HNSCC exclusively, and Wise-Draper et al. [14] enrolled a population including p16-negative oropharyngeal disease. Numbers within HPV subgroups are small, response definitions differ across these trials, and no perioperative study has been powered for a formal HPV-by-treatment interaction. The prudent conclusion is that an HPV-related difference in pathologic response is plausible and partially supported but not established.

Whether the predictive value of PD-L1 CPS is consistent across HPV subgroups is likewise unresolved. CPS functions as a stratification framework in KEYNOTE-689 and underpins the CPS-based regulatory indication, but the trial was stratified by CPS rather than by CPS within HPV stratum, and the smaller perioperative cohorts that report PD-L1 associations are too small to determine whether a given CPS threshold carries the same meaning in HPV-positive and HPV-negative tumors. Because PD-L1 expression and HPV status are themselves correlated, treating CPS as an HPV-independent predictor is an assumption rather than a finding.

De-escalation considerations apply asymmetrically. In favorable-risk HPV-positive disease, where survival with standard therapy is already high and the dominant clinical problem is long-term treatment-related morbidity, the relevant question is whether immunotherapy could permit reduction of RT or cisplatin exposure rather than be added on top of it. Adding perioperative immunotherapy to full-intensity standard therapy in this group risks compounding toxicity in patients with limited room for outcome improvement, and the benefit-risk calculus differs fundamentally from that in high-risk disease. Perioperative immunotherapy should not be assumed appropriate for favorable-risk HPV-positive patients outside a trial simply because it is approved for CPS-positive resectable disease.

Conversely, HPV-negative high-risk disease plausibly represents both the greatest unmet need and the population with the most to gain from perioperative intensification. Recurrence after surgery and risk-adapted adjuvant therapy remains common, salvage options are limited, and the immunologically colder microenvironment that makes these tumors harder to treat is also the context in which neoadjuvant priming before immune disruption is most mechanistically attractive. Future perioperative trials should prospectively stratify by HPV status and report outcomes within strata; EFS, and PD-L1 associations separately by HPV status across the trials in Table 1 would substantially improve the clinical utility of cross-trial synthesis, and we recommend this as a standard for future reporting.

## Biomarkers and response stratification in perioperative immunotherapy

PD-L1 expression, most commonly assessed as CPS, shows a modest association with perioperative PD-1 response in several cohorts. In neoadjuvant pembrolizumab studies, higher CPS has been associated with greater likelihood or depth of pathologic regression, while meaningful responses still occur in PD-L1-low tumors, limiting PD-L1 CPS as a stand-alone stratifier [13, 14]. In the phase III KEYNOTE-689 trial, EFS benefit with perioperative pembrolizumab was observed across CPS strata, with numerically greater benefit in CPS ≥ 10, supporting CPS primarily as an enrichment framework rather than an absolute determinant of benefit [10].

Beyond PD-L1 staining, immune-inflamed gene-expression programs, often characterized by IFN-γ-related signaling, chemokines, and cytotoxic T-cell transcriptional features, have been repeatedly reported as correlates of pathologic regression in perioperative HNSCC datasets [13, 14]. These profiles likely capture a broader immune activation state than PD-L1 alone and align with the “inflamed” versus “immune-excluded/cold” phenotype framework used across tumor types.

Single-cell and spatial profiling has further refined this paradigm by implicating specific intratumoral T-cell states. In resectable HNSCC treated with neoadjuvant anti-PD-1, response has been associated with activation of pre-existing cytotoxic, tissue-resident memory-like CD8^+^ T-cell programs (e.g., CTX^+^/ZNF683^+^), while clonal replacement and revival of non-exhausted persisting clones were described as relatively modest [30]. This supports a mechanistic model in which perioperative PD-1 blockade preferentially amplifies pre-existing antitumor immunity in tumors that already contain cytotoxic resident T-cell populations.

Conversely, poor pathologic response has been associated with features of an immunosuppressive tumor microenvironment, including myeloid-skewed inflammation, hypoxia-linked transcriptional programs, and compensatory upregulation of alternative immune checkpoints in non-responders in some datasets [13, 14, 30]. These correlative patterns provide biologic rationale for combination approaches targeting myeloid programs, hypoxia, or additional checkpoints, but should be framed as hypothesis-generating rather than definitive resistance mechanisms.

Liquid biopsy approaches offer minimally invasive tools for response monitoring and postoperative surveillance. In HPV-associated oropharyngeal squamous cell carcinoma, circulating HPV DNA (ctHPV-DNA) is increasingly described as a promising marker for diagnosis, treatment response monitoring, and minimal residual disease (MRD)/surveillance applications [38]. However, interpretation of ctHPV-DNA kinetics requires caution, because circulating analyte levels may be influenced by tumor burden, biologic variability in shedding and clearance, assay platform, sample source, and pre-analytic handling, with lower sensitivity in some low-volume settings [38]. Accordingly, liquid biopsy is best viewed as a complementary systemic surveillance tool rather than a standalone localizing test: a persistent or rising ctHPV-DNA signal may suggest residual or recurrent disease, but anatomic correlation with imaging and, when feasible, site-directed tissue biopsy remains important for confirming the site and histologic context of recurrence [38]. More proximal compartment-specific sampling, such as postoperative lymphatic drainage, may improve sensitivity in selected settings, but these approaches remain investigational [38]. Integration of ctHPV-DNA kinetics into perioperative immunotherapy decision-making therefore remains an area of active study, and analogous MRD tools for HPV-negative HNSCC are less mature [38].

T-cell receptor (TCR) repertoire features are an emerging class of translational biomarkers. Across cancers, TCR diversity and clonality of tumor-infiltrating lymphocytes have been linked to prognosis and, in some settings, response to PD-1 blockade [39, 40], and methodological frameworks for TCR sequencing are increasingly standardized [41]. In neoadjuvant HNSCC, single-cell transcriptomic and TCR profiling has shown that distinct CD8^+^ T-cell states and clonal dynamics can associate with pathologic response, with regimen-specific effects on effector/memory and tissue-resident programs [30, 42]. These approaches remain research-grade but may ultimately complement tissue and circulating biomarkers.

Because these markers are frequently discussed together, it is worth stating plainly which are clinically actionable today and which are not. Only two are actionable at present. PD-L1 CPS is the sole biomarker with a regulatory role in this setting: it is measured by a validated companion assay, was used for stratification in KEYNOTE-689, and defines the CPS ≥ 1 population for which perioperative pembrolizumab is approved. HPV/p16 status is likewise standard, reproducible, and required for staging and prognostication, although it does not currently direct perioperative immunotherapy selection. Every other marker discussed in this section is exploratory. Immune-inflamed and IFN-γ-related gene-expression signatures, tissue-resident cytotoxic CD8^+^ T-cell states identified by single-cell or spatial profiling, ctHPV-DNA kinetics, and TCR repertoire dynamics all show associations with response in selected datasets, but none has a validated threshold, a standardized assay, an established analytic pipeline, or prospective evidence that acting on the result improves outcomes. ctHPV-DNA in particular is often discussed as though it were ready for clinical use; it is not yet validated for perioperative treatment decisions, and its role in escalation or de-escalation remains a trial question rather than a practice standard. These exploratory markers should inform trial design and hypothesis generation, and should not be used to select, escalate, de-escalate, or discontinue perioperative immunotherapy outside a protocol.

## Surgical feasibility, toxicity, and multidisciplinary considerations

Early neoadjuvant PD-1 studies were designed around feasibility endpoints (including protocol-defined surgical delays) and generally demonstrate that short-course preoperative therapy can be delivered without routinely preventing curative-intent resection [13–15]. In the phase III KEYNOTE-689 trial, surgical completion rates were similar between arms (88.4% with perioperative pembrolizumab vs. 87.7% control); however, surgery delays were more frequent in the neoadjuvant pembrolizumab arm (38 vs. 10 participants), underscoring that perioperative immunotherapy can introduce measurable scheduling and recovery friction even when resection remains achievable.

Curative-intent outcomes in HNSCC depend on timely, risk-adapted delivery of postoperative RT or CRT. In KEYNOTE-689, postoperative RT/CRT was recommended to begin after adequate recovery (≤ 6 weeks), and only a subset initiated adjuvant therapy within that timeframe (46.4% pembrolizumab vs. 34.8% control; most started within 8 weeks). These data highlight a practical point for real-world implementation: perioperative protocols should be built to protect the postoperative RT/CRT window (e.g., predefined infusion scheduling, early toxicity triage pathways, and explicit criteria for holding immunotherapy when wound or systemic issues threaten adjuvant start).

Perioperative PD-1 toxicity generally follows established class patterns, but its significance in the perioperative setting is amplified by competing priorities: surgical recovery, nutrition/swallow function, and readiness for adjuvant RT/CRT. Accordingly, feasibility in trials should be interpreted as “deliverable with safeguards,” rather than “toxicity-neutral,” particularly when adjuvant immunotherapy is prolonged (e.g., KEYNOTE-689 postoperative pembrolizumab integrated with RT/CRT and continued adjuvantly).

Functional outcomes deserve greater prominence than they have generally received in this literature. In HNSCC, cure is not the only endpoint that matters to patients: swallowing function, feeding-tube dependence, speech, airway, shoulder function, and overall quality of life determine much of the lived result of treatment, and the anatomy involved means that functional loss is often permanent and highly visible. These outcomes are central to how patients and multidisciplinary teams weigh treatment intensification, yet they are inconsistently captured across perioperative immunotherapy trials, rarely reported as primary or prespecified secondary endpoints, and typically followed for shorter intervals than survival endpoints. The consequence is that the functional cost of adding perioperative immunotherapy to surgery and adjuvant RT/CRT is currently unquantified. This gap matters in two directions. Immune-related toxicity, wound complications, or delays to postoperative RT/CRT could plausibly worsen swallowing and feeding-tube outcomes, and prolonged adjuvant immunotherapy layered onto CRT adds a symptom burden during precisely the period when nutritional status and swallowing rehabilitation are most fragile. Conversely, if pathologic response were eventually shown to permit de-intensification of adjuvant therapy, the principal benefit might be functional rather than survival-based. Neither possibility can currently be evaluated. Routine, prespecified capture of validated functional and patient-reported outcome measures, reported at fixed intervals alongside EFS, should be considered a requirement rather than an optional addition in future perioperative trials.

Concerns are most acute for patients requiring complex reconstruction (including free flaps) and for salvage settings with previously irradiated tissues. Retrospective evidence is heterogeneous. In a multi-institutional AHNS reconstructive microsurgery committee series, recipient-site wound complications occurred in 31% and major complications in 14%; preoperative immunotherapy exposure was associated with increased odds of major complications (OR 3.7) and need for treatment of wound complications (OR 2.9). In contrast, a separate retrospective cohort with propensity matching comparing surgery with versus without neoadjuvant ICI reported similar overall complication rates after matching and did not identify neoadjuvant ICI as an independent driver of complications on regression modeling. These conclusions are discordant, and the divergence is most plausibly methodological rather than biological. The two datasets differ in several respects that could each generate the observed difference: the reconstructive microsurgery series was assembled specifically from free-flap cases and therefore enriched for complex, high-risk reconstruction, whereas the propensity-matched cohort included a broader surgical case mix in which flap complexity was diluted; prior radiation burden and salvage status, which are among the strongest determinants of wound healing, were distributed differently and only partially adjusted for; institutional volume and reconstructive expertise varied across contributing centers in the multi-institutional series; and the propensity-matching approach, while balancing measured covariates, cannot address unmeasured confounding by indication, since patients selected for neoadjuvant ICI may differ systematically in nutritional status, smoking, and comorbidity from those taken directly to surgery. Outcome ascertainment also differed, with no shared definition of what constituted a major wound complication. Until surgical endpoints are captured prospectively and uniformly within perioperative trials, neither the positive nor the null finding should be applied as a clinical rule; the honest summary is that a wound-complication signal cannot be excluded, particularly in free-flap and salvage settings, and should be discussed with patients and reconstructive colleagues on a case-by-case basis [43].

Because HNSCC treatment planning is intrinsically multimodal, perioperative immunotherapy should be operationalized through structured, multidisciplinary pathways rather than ad hoc additions to existing schedules. NCCN guidance emphasizes that initial evaluation and plan development requires a multidisciplinary team, and that managing sequelae after surgery, RT, and systemic therapy requires coordinated supportive care and rehabilitation. For perioperative immunotherapy, this translates into (1) shared “go/no-go” criteria for proceeding to surgery, (2) early postoperative wound and swallow surveillance, (3) predefined management for immune-related toxicities that could delay RT/CRT, and (4) clear communication between surgical oncology, medical oncology, radiation oncology, pathology, and supportive-care services.

In salvage populations, where prior RT and comorbidity burden are common, perioperative immunotherapy has been feasible in trial settings without routine operative delay but remains particularly sensitive to wound and recovery issues [20, 21].

## Practical implementation questions

Several questions arise immediately in clinic and are not resolved by the trial data; they are set out here explicitly, with the evidence gaps named rather than papered over.

CPS-negative patients. The FDA indication is restricted to CPS ≥ 1, yet CPS-negative patients were randomized in KEYNOTE-689 and the trial demonstrated EFS benefit in the total population. Off-indication use in CPS-negative disease is not supported by regulatory approval and should not be routine; these patients are reasonable candidates for clinical trials, and treatment decisions should proceed through standard surgery and risk-adapted adjuvant therapy outside a protocol.

Favorable-risk HPV-positive disease. As discussed above, the benefit-risk balance in this group differs from that in high-risk disease, and perioperative immunotherapy should not be extrapolated to favorable-risk HPV-positive patients on the basis of a CPS-based indication alone. De-escalation questions in this population belong in trials.

irAEs that threaten postoperative RT/CRT. Because timely adjuvant therapy is an established determinant of cure, irAEs in the perioperative window carry consequences beyond their own severity. Programs should predefine which toxicities trigger holding immunotherapy versus delaying RT/CRT, establish rapid-access pathways to medical oncology for grading and steroid initiation, and treat the adjuvant start window as a protected target rather than a flexible one. In general, low-grade irAEs should be managed without delaying RT/CRT, whereas events requiring systemic corticosteroids warrant explicit multidisciplinary decision-making about sequencing.

Free flap reconstruction. Given the discordant retrospective data discussed above, patients requiring complex free flap reconstruction, and particularly those undergoing salvage surgery in previously irradiated fields, warrant individualized discussion in which a possible but unproven wound-complication signal is disclosed. Reconstructive surgeons should be involved before neoadjuvant immunotherapy is committed to, not after.

Frail patients and autoimmune contraindications. Perioperative trials enrolled patients fit for major oncologic surgery followed by cisplatin-based CRT, and largely excluded active autoimmune disease and significant immunosuppression. The evidence base therefore says little about frail patients or those with autoimmune contraindications, and benefit should not be assumed to extend to them; in this group the added toxicity of prolonged adjuvant immunotherapy may outweigh an unquantified benefit.

Real-world applicability. Perioperative immunotherapy in HNSCC is a systems intervention as much as a pharmacologic one. It presumes rapid pathology turnaround, coordinated infusion and operative scheduling, available CPS testing, on-call toxicity triage, and reconstructive and supportive care infrastructure. These conditions characterize the high-volume academic centers where the trials were conducted and may not be reproducible elsewhere. Centers without this infrastructure should weigh the risk that delays to surgery or to postoperative RT/CRT erode the very benefit the strategy is intended to deliver, and referral to a high-volume center may be more appropriate than local adoption.

## Limitations

Several limitations should be considered when interpreting perioperative immunotherapy evidence in resectable HNSCC. First, the literature remains highly heterogeneous across trial phase and design (single-arm vs. randomized), patient selection, tumor subsite distribution, HPV/p16 enrichment, risk stratification schemas, and treatment backbones (neoadjuvant-only vs. neoadjuvant-adjuvant, postoperative RT vs. CRT, and salvage settings). This heterogeneity extends to endpoint definitions and reporting, including variable pathologic response frameworks (pTR categories vs. MPR/pCR thresholds; central vs. local pathology review), inconsistent attribution windows for irAEs, and non-uniform definitions of “surgical delay” (protocol-defined delay vs. any postponement). As a result, direct cross-trial comparisons are inherently limited, and formal quantitative pooling is often inappropriate even when studies appear conceptually similar.

Second, evidence maturity varies substantially by strategy. Many perioperative regimens are supported primarily by early-phase or exploratory datasets with modest sample sizes and limited long-term follow-up. While event-free and disease-free outcomes are increasingly reported, OS data and durable functional outcomes (swallowing, quality of life, long-term feeding-tube dependence) remain immature for several approaches. Moreover, the degree to which early pathologic endpoints generalize as validated surrogates for long-term survival in HNSCC is still incompletely defined, particularly across distinct etiologic subgroups (HPV-positive vs. HPV-negative disease) and across divergent perioperative platforms (monotherapy vs. combination regimens).

Third, “feasibility” should be interpreted as deliverability within structured protocols rather than absence of downstream consequences. Even when surgical completion is preserved, perioperative immunotherapy can introduce practical scheduling and recovery friction that may affect the timing of postoperative RT/CRT, an established determinant of curative-intent outcomes and a key systems constraint emphasized throughout this review. Feasibility signals are also influenced by selection effects (patients fit for major oncologic surgery, often treated at high-volume centers with multidisciplinary infrastructure), which may limit generalizability to resource-variable settings.

Fourth, surgical wound and reconstruction data are largely retrospective and confounded. Available flap and wound complication series are heterogeneous, and differences in baseline risk (nutritional status, smoking, diabetes, prior RT, flap complexity, salvage operations) can strongly influence observed outcomes. Consequently, signals suggesting increased wound complications with neoadjuvant ICI in some cohorts should be interpreted cautiously, and prospective, protocolized capture of surgical endpoints is needed before firm causal inferences can be made.

Fifth, biomarker evidence remains predominantly exploratory. PD-L1 CPS, immune-inflamed gene-expression programs, tissue-resident T-cell states, ctHPV-DNA, and TCR dynamics show promising associations in selected datasets, but assays, thresholds, sampling timepoints, and analytic pipelines are not standardized, and access to high-dimensional profiling varies widely across institutions. Tissue availability (biopsy size, spatial heterogeneity), batch effects, and limited external validation further constrain the clinical actionability of these markers at present.

Finally, as a narrative review, this work is subject to inherent selection and publication biases and does not include a protocol-driven risk-of-bias appraisal. Some emerging strategies are supported by interim reports or evolving trial datasets, which may shift as follow-up matures. Ongoing randomized trials, standardized reporting of surgical and functional endpoints, and prospective biomarker-driven investigations will be essential to refine perioperative immunotherapy strategies and define their optimal integration into curative-intent management of resectable HNSCC.

## Future directions

The emergence of perioperative immune checkpoint inhibition as an effective strategy in resectable HNSCC represents a meaningful advance in curative-intent management, but the field now shifts from “does it work” to how to optimize who receives it, when, and for how long. Future work should prioritize trial designs and implementation frameworks that preserve oncologic standards (particularly postoperative RT/CRT timing) while enabling biology-driven personalization.

A near-term priority is harmonization of perioperative endpoints to enable defensible cross-trial interpretation. This includes standardized pathologic response definitions (and central review when feasible), explicit definitions of “surgical delay” (protocol-defined vs. any postponement), consistent attribution windows for immune-related toxicity, and routine capture of surgery-relevant outcomes (wound complications, flap outcomes, unplanned returns to OR) alongside functional endpoints (swallowing, feeding-tube dependence, quality of life). These measures should be reported with sufficient granularity to distinguish feasibility within protocolized trial infrastructure from real-world deliverability.

Biomarker development should move from retrospective association to prospective decision utility. PD-L1 CPS, immune-inflamed transcriptional programs, and tissue-resident cytotoxic T-cell states provide plausible stratification frameworks, but clinically actionable thresholds and reproducibility across assays must be established. Trials should embed standardized biospecimen collection and prespecified biomarker hypotheses, and incorporate dynamic monitoring tools (e.g., circulating tumor DNA/ctHPV-DNA where applicable and immune repertoire readouts) to test response-adapted postoperative strategies rather than relying on static baseline markers alone.

The relative contributions of neoadjuvant priming versus prolonged adjuvant immunotherapy remain incompletely defined, particularly when layered onto postoperative RT/CRT. Future trials should explicitly test duration minimization and sequencing questions (e.g., shorter adjuvant courses, response-adapted continuation/cessation, and regimen timing relative to RT/CRT) to balance durable disease control against cumulative toxicity and treatment burden. Where pathologic or molecular response is robust, de-escalation strategies should be studied prospectively; conversely, non-responders may require rational escalation rather than prolonged monotherapy.

Mechanism-informed combination strategies (e.g., targeting myeloid inflammation, hypoxia-linked programs, alternative checkpoints, or immunometabolic pathways) should be pursued in biomarker-enriched populations most likely to benefit, but with perioperative safety as a primary constraint. Window-of-opportunity platforms remain valuable for rapidly testing biologic activity and resistance mechanisms, but curative-intent adoption should depend on randomized confirmation that combinations improve outcomes without compromising surgical recovery or postoperative RT/CRT delivery.

As perioperative immunotherapy expands beyond trial settings, standardized multidisciplinary pathways will be essential. Practical frameworks should coordinate imaging, infusion scheduling, surgery, pathology turnaround, toxicity triage, and postoperative RT/CRT start targets, while supporting equitable access to biomarker assays and high-quality supportive care. Real-world evaluations should measure not only survival outcomes, but also treatment timeliness, functional recovery, and complication burden, because the success of perioperative immunotherapy ultimately depends on systems performance as much as biologic activity.

### Clinical take-home points

#### What the evidence currently supports

Perioperative pembrolizumab added to surgery and risk-adapted adjuvant therapy improves EFS in patients with resectable, locally advanced HNSCC, based on the positive findings of the phase III KEYNOTE-689 trial. However, OS data remain immature. The FDA approval issued in June 2025 applies specifically to tumors with a PD-L1 CPS of at least 1. Because the trial population and the approved indication are not identical, CPS testing is required before treatment initiation. Importantly, the available evidence supports this specific pembrolizumab-based regimen rather than perioperative immune checkpoint inhibition as a class effect. Other approaches, including dual checkpoint blockade and perioperative chemoimmunotherapy, remain investigational.

#### What the evidence does not establish

The KEYNOTE-689 design does not establish which component of the treatment strategy is responsible for the observed benefit. The relative contributions of neoadjuvant immune priming, concurrent postoperative therapy, and prolonged adjuvant maintenance cannot be separated, and no schedule can yet be considered optimal. The evidence also does not establish pathologic response as a surrogate for survival. pCR and major pathologic response remain nonstandardized in HNSCC and have not been validated as surrogate endpoints; therefore, they should not guide treatment decisions outside of clinical trials. Similarly, biomarkers beyond CPS and HPV status are not ready for routine clinical use. Gene-expression signatures, ctHPV-DNA, and TCR dynamics remain exploratory.

#### Practice points

Protection of the postoperative RT or CRT window should remain a priority. Timely delivery of adjuvant therapy is an established determinant of cure, and institutions should predefine toxicity-triage pathways and treatment-holding criteria to ensure that immunotherapy does not delay or displace definitive postoperative treatment. Surgery remained feasible in KEYNOTE-689, with surgical completion rates of 88.4% in the pembrolizumab group and 87.7% in the control group, although surgical delays were more frequent with pembrolizumab, occurring in 38 patients compared with 10 patients in the control group. Patients should be counseled regarding this possibility.

Early involvement of reconstructive surgery is also advisable, particularly for patients undergoing free-flap reconstruction or salvage surgery, because a wound-complication signal associated with neoadjuvant immune checkpoint inhibition cannot yet be excluded. More broadly, implementation of perioperative immunotherapy should be viewed as a systems-level intervention. Without timely pathology reporting, coordinated surgical and oncology scheduling, and appropriate toxicity-triage infrastructure, the benefits demonstrated in clinical trials may not be reproducible in routine practice. In settings where these resources are unavailable, referral to an experienced multidisciplinary center may be preferable to local adoption.

## Conclusions

Current evidence supports perioperative pembrolizumab added to surgery and risk-adapted adjuvant therapy as an effective strategy in selected patients with resectable locally advanced HNSCC, while other perioperative ICI regimens remain investigational. This conclusion should be stated at the level the evidence supports. Across early-phase window-of-opportunity studies, perioperative PD-1-based strategies demonstrated biologic activity and preserved surgical deliverability; definitive evidence of clinical benefit, however, rests on a single positive randomized phase III trial (KEYNOTE-689), in which EFS improved when perioperative pembrolizumab was integrated with surgery and risk-adapted postoperative therapy, and in which OS data were not mature. The corresponding FDA approval is restricted to disease with a PD-L1 CPS of at least 1. A single trial of one agent is a narrow evidentiary base from which to generalize to a therapeutic class, and it should not be read as establishing perioperative immune checkpoint inhibition as broadly effective. These results contrast with the largely negative experience of adding PD-1/PD-L1 blockade to definitive CRT in unresected locally advanced disease and reinforce that treatment timing and immune context are central determinants of benefit in HNSCC. Mechanistic and translational data support a model in which neoadjuvant checkpoint blockade leverages an intact tumor-immune interface and tumor-draining immune architecture to amplify tumor-reactive T-cell programs prior to definitive resection, while postoperative immunotherapy may help sustain immune pressure during a period of heightened recurrence risk. Emerging biomarker work further suggests that response and benefit may be enriched in immune-inflamed states and specific cytotoxic T-cell programs, offering a foundation for future response-adapted perioperative strategies; with the exception of PD-L1 CPS and HPV status, however, these markers remain exploratory and are not yet appropriate for treatment selection outside a protocol. The relative contributions of neoadjuvant priming, concurrent postoperative therapy, and prolonged adjuvant maintenance cannot be disentangled within the available trial design, and no perioperative sequence can presently be designated optimal. As perioperative immunotherapy moves toward broader adoption, the next phase of progress will depend on refining sequencing and duration, developing combination approaches for resistant phenotypes without widening perioperative risk, and prospectively validating biomarker frameworks that are reproducible and clinically actionable.

## Abbreviations

AE: adverse event
CI: confidence interval
CPS: combined positive score
CRT: chemoradiotherapy
ctHPV-DNA: circulating human papillomavirus DNA
CTLA-4: cytotoxic T-lymphocyte-associated antigen 4
DFS: disease-free survival
DLT: dose-limiting toxicity
EFS: event-free survival
GEJ: gastroesophageal junction
HNSCC: head and neck squamous cell carcinoma
HPV: human papillomavirus
HR: hazard ratio
ICIs: immune checkpoint inhibitors
IFN-γ: interferon-gamma
irAEs: immune-related adverse events
LA: locally advanced
MPR: major pathologic response
MRD: minimal residual disease
NSCLC: non-small cell lung cancer
ORR: objective response rate
OS: overall survival
pCR: pathologic complete response
PD-1: programmed cell death 1
PD-L1: programmed death-ligand 1
PFS: progression-free survival
pPR: pathologic partial response
pTR: pathologic tumor response
q2w: every 2 weeks
q3w: every 3 weeks
RT: radiotherapy
SCC: squamous cell carcinoma
SOC: standard of care
TCR: T-cell receptor
TRAE: treatment-related adverse event

## Declarations

### Copyright

© The Author(s) 2026.
